# Activation of Erk and JNK MAPK pathways by acute swim stress in rat brain regions

**DOI:** 10.1186/1471-2202-5-36

**Published:** 2004-09-20

**Authors:** Chang-peng Shen, Yelena Tsimberg, Christopher Salvadore, Emanuel Meller

**Affiliations:** 1 Department of Psychiatry, New York University School of Medicine, 550 First Ave, MHL HN511, New York, NY 10016, USA; 2 Cell Signaling Technology, 166B Cummings Center, Beverly, MA 09115, USA

## Abstract

**Background:**

The mitogen-activated protein kinases (MAPKs) have been shown to participate in a wide array of cellular functions. A role for some MAPKs (e.g., extracellular signal-regulated kinase, Erk1/2) has been documented in response to certain physiological stimuli, such as ischemia, visceral pain and electroconvulsive shock. We recently demonstrated that restraint stress activates the Erk MAPK pathway, but not c-Jun-N-terminal kinase/stress-activated protein kinase (JNK/SAPK) or p38MAPK, in several rat brain regions. In the present study, we investigated the effects of a different stressor, acute forced swim stress, on the phosphorylation (P) state of these MAPKs in the hippocampus, neocortex, prefrontal cortex, amygdala and striatum. In addition, effects on the phosphorylation state of the upstream activators of the MAPKs, their respective MAPK kinases (MAPKKs; P-MEK1/2, P-MKK4 and P-MKK3/6), were determined. Finally, because the Erk pathway can activate c-AMP response element (CRE) binding (CREB) protein, and swim stress has recently been reported to enhance CREB phosphorylation, changes in P-CREB were also examined.

**Results:**

A single 15 min session of forced swimming increased P-Erk2 levels 2–3-fold in the neocortex, prefrontal cortex and striatum, but not in the hippocampus or amygdala. P-JNK levels (P-JNK1 and/or P-JNK2/3) were increased in all brain regions about 2–5-fold, whereas P-p38MAPK levels remained essentially unchanged. Surprisingly, levels of the phosphorylated MAPKKs, P-MEK1/2 and P-MKK4 (activators of the Erk and JNK pathways, respectively) were increased in all five brain regions, and much more dramatically (P-MEK1/2, 4.5 to > 100-fold; P-MKK4, 12 to ~300-fold). Consistent with the lack of forced swim on phosphorylation of p38MAPK, there appeared to be no change in levels of its activator, P-MKK3/6. P-CREB was increased in all but cortical (prefrontal, neocortex) areas.

**Conclusions:**

Swim stress specifically and markedly enhanced the phosphorylation of the MAPKKs P-MEK1/2 and P-MKK4 in all brain regions tested without apparent alteration in the phosphorylation of P-MKK3/6. Curiously, phosphorylation of their cognate substrates (Erk and JNK) was increased to a much more modest extent, and in some brain regions was not altered. Similarly, there was a region-specific discrepancy between Erk and CREB phosphorylation. Possible explanations for these findings and comparison with the effects of restraint stress will be discussed.

## Background

Increasing evidence implicates stress as an important factor in the vulnerability to depressive and other mental illnesses [1-3]. Consequently, understanding the cellular and biochemical mechanisms that transduce stress signals may inform our insight into the factors that lead to depression, as well as provide potential new avenues for therapeutic intervention. Although current therapies for anxiety and depression usually focus on modulating serotonergic and/or noradrenergic activity in the brain [4], recent data suggest that neurotrophins such as BDNF may ameliorate depression [5]. Neurotrophins activate MAPK signaling, and increased signaling *via *at least one of the MAPKs, Erk, has been shown to regulate plasticity in the brain by modulating gene expression. Thus, activation of the Erk pathway *via *both a cAMP/PKA-dependent mechanism leading to activation of CREB, and a non-cyclase-linked pathway, leading to activation of CREB and/or the serum response element (SRE) binding protein Elk-1, have been shown to occur in the hippocampus during acquisition of learning, memory consolidation and long-term potentiation (LTP) [6-8]. It is therefore not unreasonable to speculate that these signal transduction pathways may also be involved in the neuronal plasticity that is presumed to underlie aberrant brain function [9].

The term MAPK is used to denote a family of signal transduction mediators, extensively distributed throughout the central nervous system [10,11], that regulate a diverse array of cellular functions [12-15]. The most common MAPKs are the extracellular signal-regulated kinases Erk1 and 2 (also known as p44 and p42MAPK, respectively), which primarily regulate cellular growth and differentiation, and the p38MAPK and c-Jun-N-terminal kinase/stress-activated protein kinases (JNK/SAPK), which mainly function as mediators of cellular stresses such as inflammation and apoptosis. Physiologically stressful stimuli, including seizure induction [16], ischemic insult [17], visceral pain [18] and electroconvulsive shock (ECS) [19,20] have also been shown to rapidly activate MAPKs in various brain regions.

Subjecting rats or mice to acute stressors such as restraint or forced swim results in activation of *c-fos *gene and/or Fos protein expression in many brain regions) [21-24]. CRE and SRE are DNA sequences found in the promoter regions of many immediate early genes (IEGs) such as *c-fos *and *zif286*. P-CREB and P-Elk-1, by binding to these elements, enhance the transcription of the IEGs and regulate gene expression [25,26]. Recently it was shown that swim stress increased P-CREB expression in several brain regions [27]. Thus, given that, on one hand, stress activated CREB and increased *c-fos *expression, and on the other, that certain stressful stimuli (see above) activated Erk, it was reasonable to predict that acute physiological stressors would activate the Erk (and perhaps other) MAPK pathway(s). The brain regions most likely to show such an effect would be those implicated in the response to stress, including the hippocampus [28,29], prefrontal [30-32] and other cortical areas [21,33], and the amygdala [21,34-36]. In a previous study we tested this hypothesis and found indeed that acute restraint stress increased P-Erk concentrations in several stress-relevant brain areas; however, neither JNK nor p38MAPK phosphorylation were altered by restraint stress [37]. The present study followed up that investigation by examining the effects of a different common stressor, forced swim stress, extended the number of brain areas examined, and also evaluated changes in the phosphorylation state of the respective upstream MAPKK activators MEK1/2, MKK4 (also known as SEK1 and JNKK) and MKK3/6. While these studies were in progress, others reported that swim stress activated the MKK4/JNK pathway in mice [38].

## Results

### Swim stress elicits large increases in phosphorylation of MEK1/2 in all brain regions

**Figure 1:**
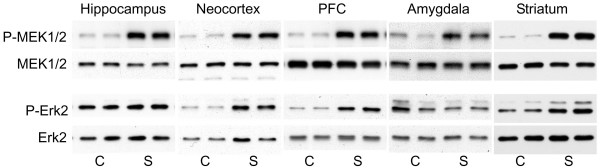
**Swim stress increased phosphorylation of MEK1/2 and Erk2 in brain. **Representative immunoblots of changes in P-MEK1/2 and P-Erk2 after subjecting rats to forced swim for 15 min. Total (pan) MEK1/2 and Erk2 were unchanged by swim stress. In this and all other immunoblot figures, molecular weight markers (not shown) corroborated the expected size of target proteins. C, control; S, swim stress.

**Figure 2:**
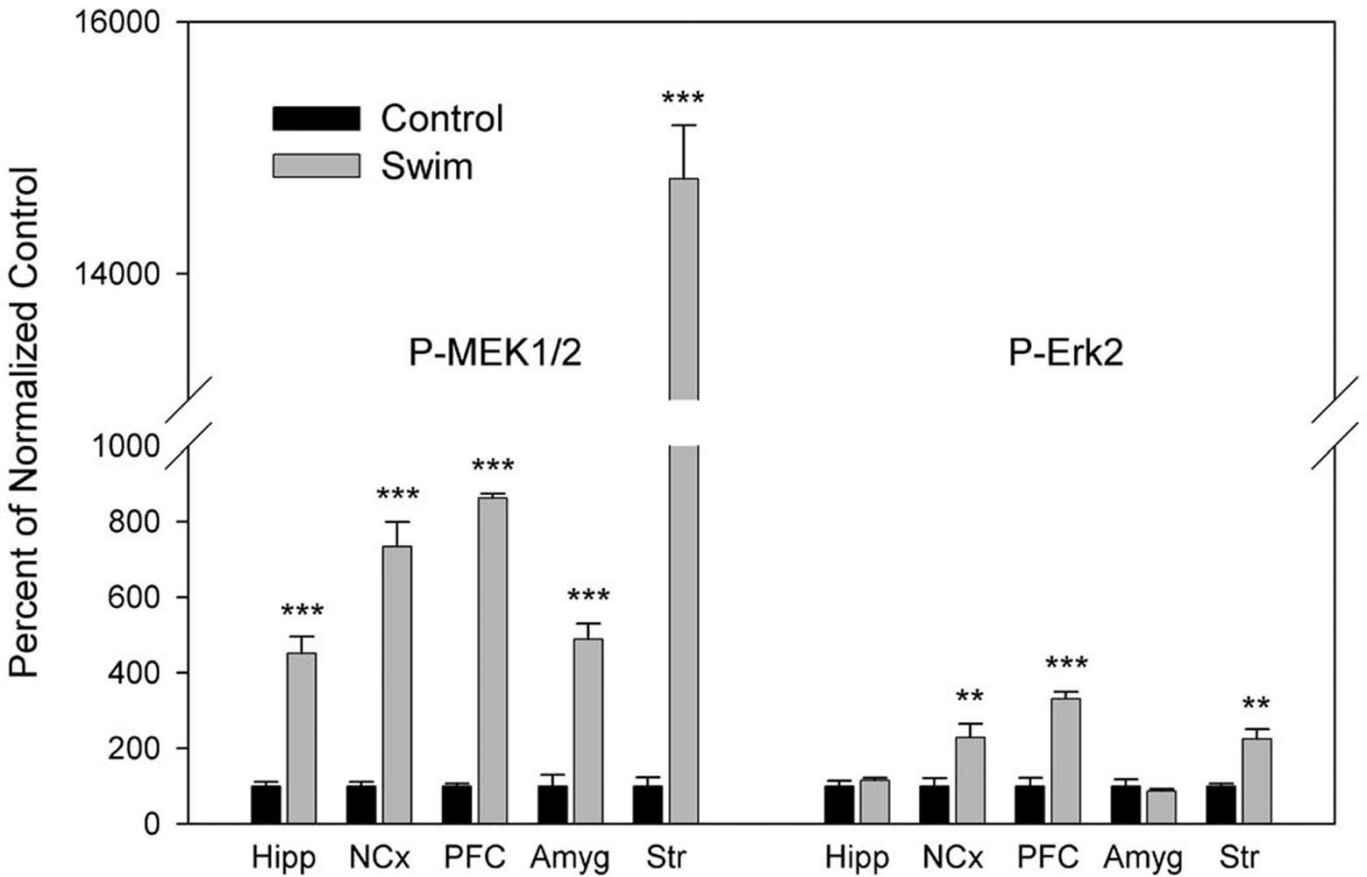
**Quantitative changes in P-MEK1/2 and P-Erk2 following swim stress. **The results are the mean ± standard error of 4–6 rats/group. * *p *< 0.05; **p < 0.01; ***p < 0.001. Hipp, hippocampus; NCx, neocortex; PFC, prefrontal cortex; Amyg, amygdala; Str, striatum.

An acute 15 min session of forced swimming produced obvious large increases in the phosphorylation of the MAPKK MEK1/2 in the hippocampus, neocortex, prefrontal cortex, amygdala and striatum (Fig. 1, top). Surprisingly, phosphorylation of the substrate(s) of activated MEK1/2, Erk1/2, was increased to a much smaller extent, and in some brain regions (hippocampus, amygdala) not at all (Fig. 1, bottom). Swim stress significantly increased P-MEK1/2 levels from 4.5-fold (hippocampus) to more than 100-fold (striatum) in the various brain areas (Fig. 2). P-Erk2 levels, on the other hand, were modestly elevated only in neocortex (2.3-fold), prefrontal cortex (3.3-fold) and striatum (2.3-fold) (Fig. 2). Because P-Erk1 signals were generally much weaker, and in short exposure blots nearly undetectable, only P-Erk2 bands were quantitated. However, qualitative changes in P-Erk1 concentrations paralleled those in P-Erk2 (Fig. 1, bottom: neocortex, amygdala, striatum); this conclusion was borne out in over-exposed blots, which, however, were difficult to quantitate for P-Erk1 because of overlap with the large adjacent P-Erk2 signal.

### Swim stress increases phosphorylation of MKK4 in all brain regions

**Figure 3:**
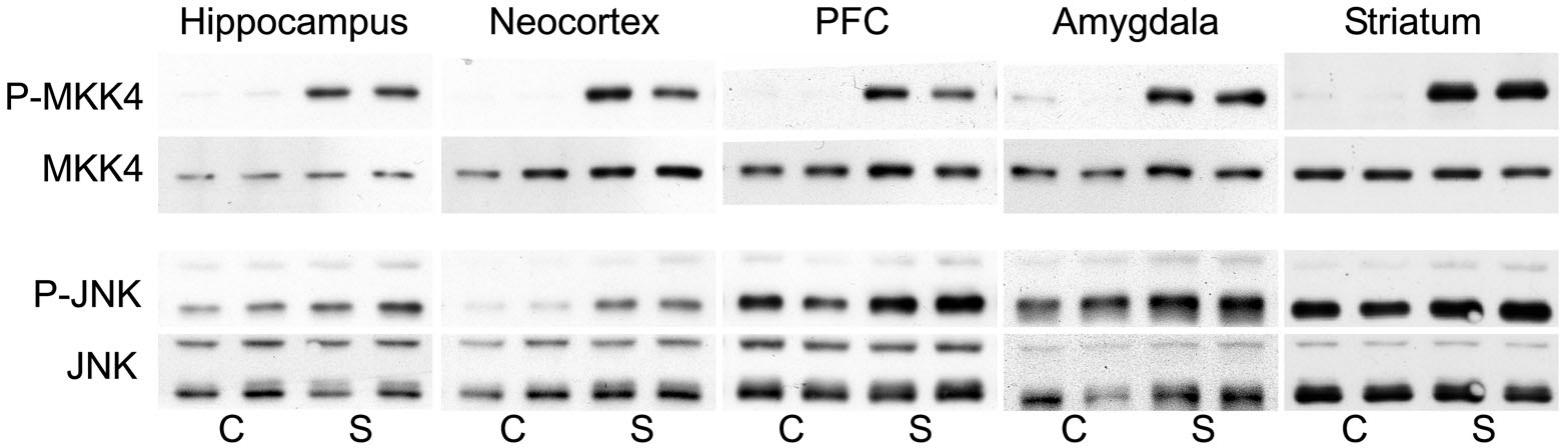
**Swim stress-induced phosphorylation of MKK4, JNK1 and JNK2/3 in brain regions. **Representative immunoblots are shown. In the JNK blots, the lower band corresponds to the 46 kD JNK1 isoform, while the upper band represents the (unseparated) 54 kD JNK2 and 3 isoforms. Abbreviations as in the legend to Fig. 1.

**Figure 4:**
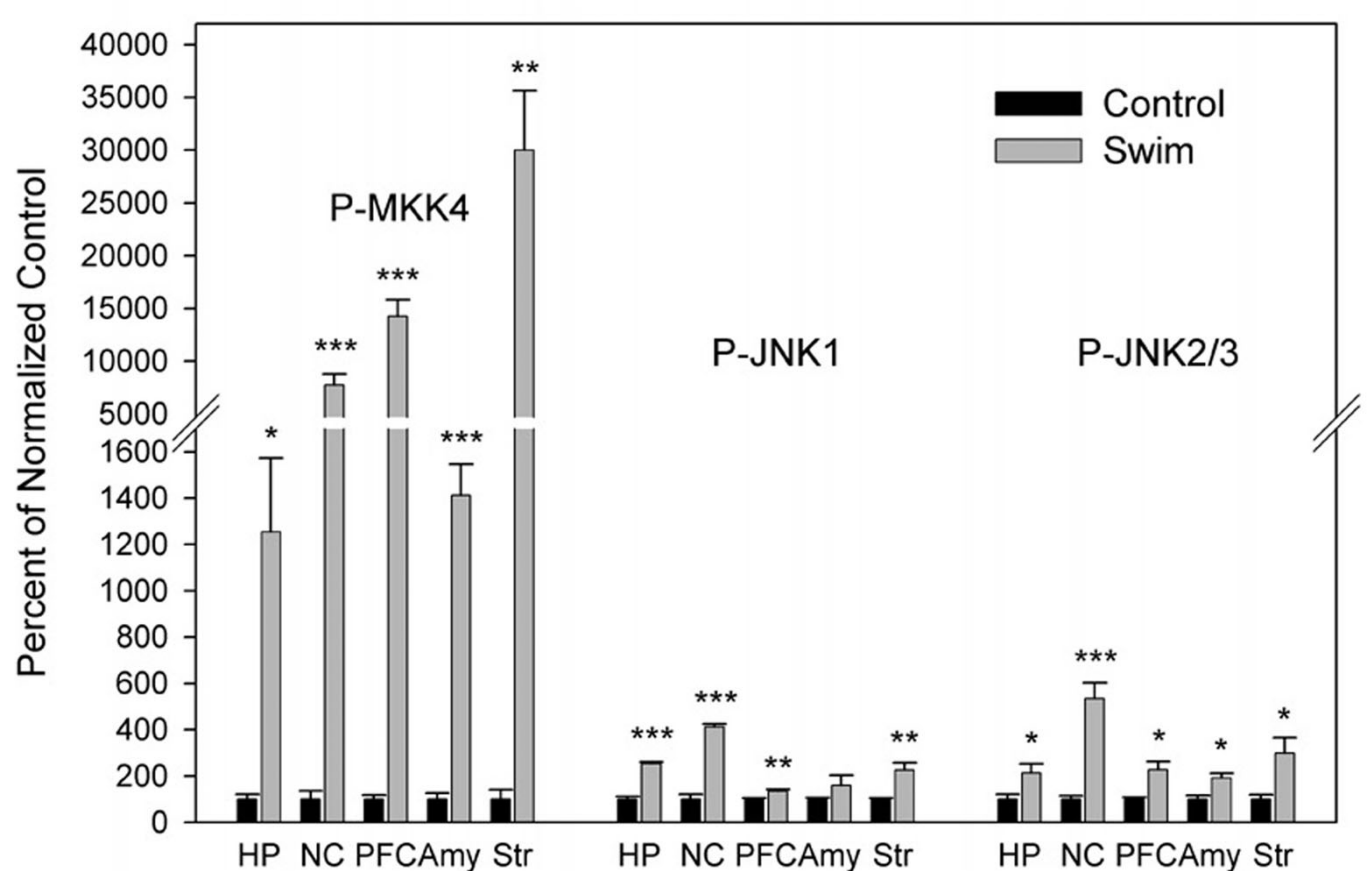
**Quantitative changes in P-MKK4 and P-JNK1 and P-JNK2/3 after swim stress. **N = 4–6 rats per group. HP, hippocampus; NC, neocortex; Amy, amygdala; other abbreviations and statistics as in the legend to Fig. 2.

Analogous to its effects on P-MEK1/2, but much more dramatically, forced swimming enhanced the levels of P-MKK4, the immediate upstream activator of JNK, in all brain areas tested (Fig. 3, top). Increases ranged from 12.6-fold (hippocampus) to ~300-fold (striatum) (Fig. 4). It should be noted that all increases greater than about 10–15-fold are approximate, since the relationship between optical density and the film exposure-response is no longer linear beyond this range, as determined previously [37]. Thus, increases greater than 10–15-fold are likely underestimates of the actual changes. Nevertheless, it seemed reasonable to utilize the calculated numerical estimates for statistical purposes. In contrast, and again analogous to the findings in the MEK/Erk pathway, much smaller changes were observed in P-JNK1 (p46) and P-JNK2/3 (p54) levels (Fig. 3, bottom). Both P-JNK1 and P-JNK2/3 were significantly elevated in all brain areas, with the single exception of a non-significant increase in P-JNK1 in the amygdala (Fig. 4). Significant swim stress-induced increases for these kinases ranged from 1.4–5.4-fold across brain regions (Fig. 4).

### Effects of swim stress on activation of the p38MAPK pathway and phosphorylation of CREB

**Figure 5:**
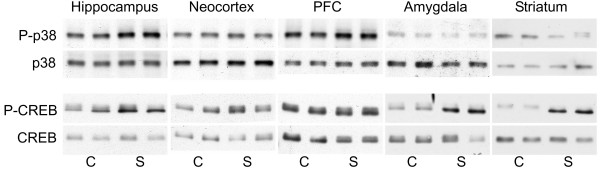
**Swim stress generally did not alter p38MAPK phosphorylation but elevated P-CREB in some brain regions. **Representative blots are shown. Abbreviations as in the legend to Fig. 1.

**Figure 6:**
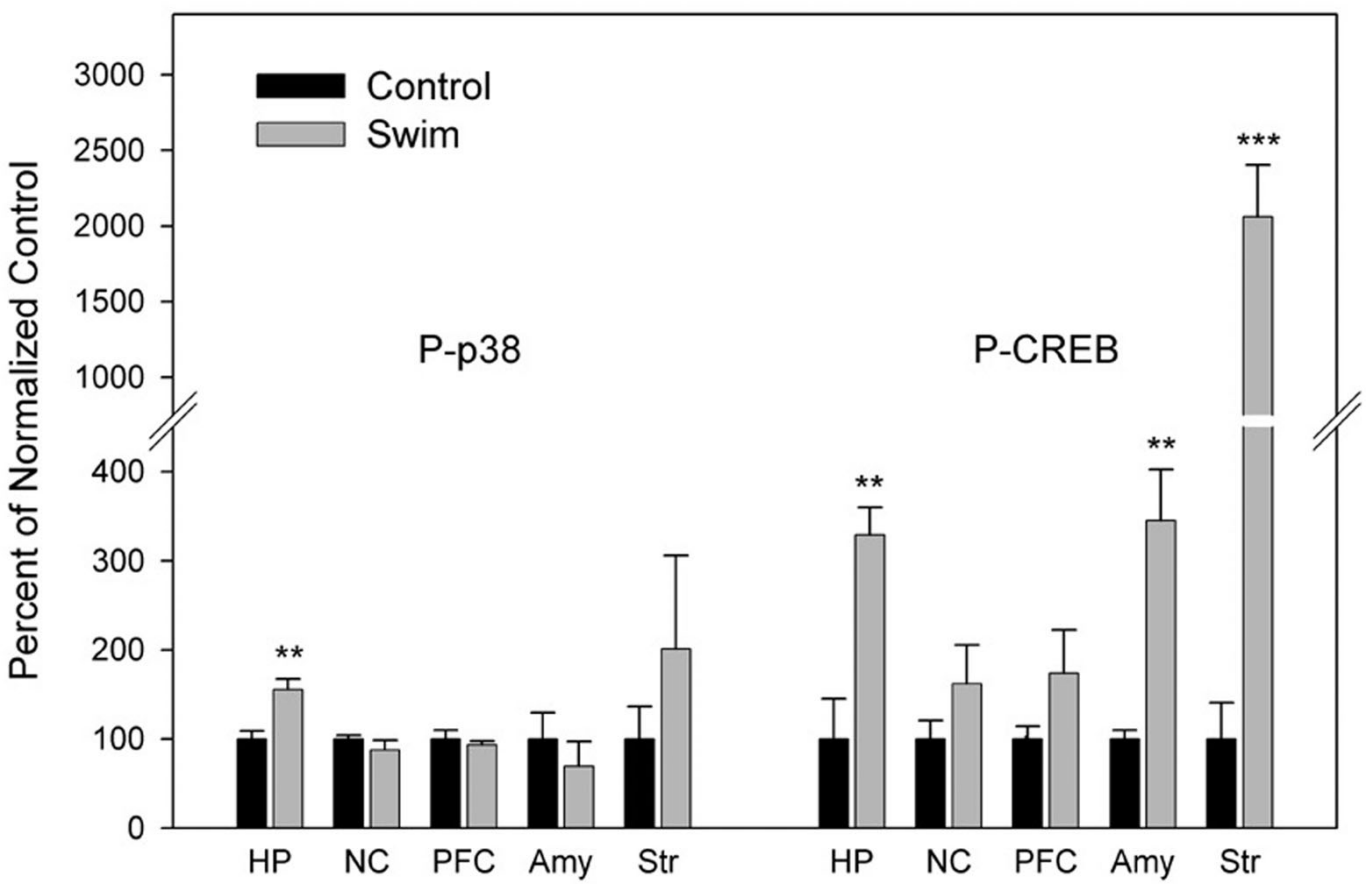
**Quantitative regional alterations in P-p38MAPK and P-CREB after swim stress. **Abbreviations as in the legends to Figs. 2 and 4; statistics as in the legend to Fig. 2. Mean ± standard error of 4–6 rats/group.

**Figure 7:**
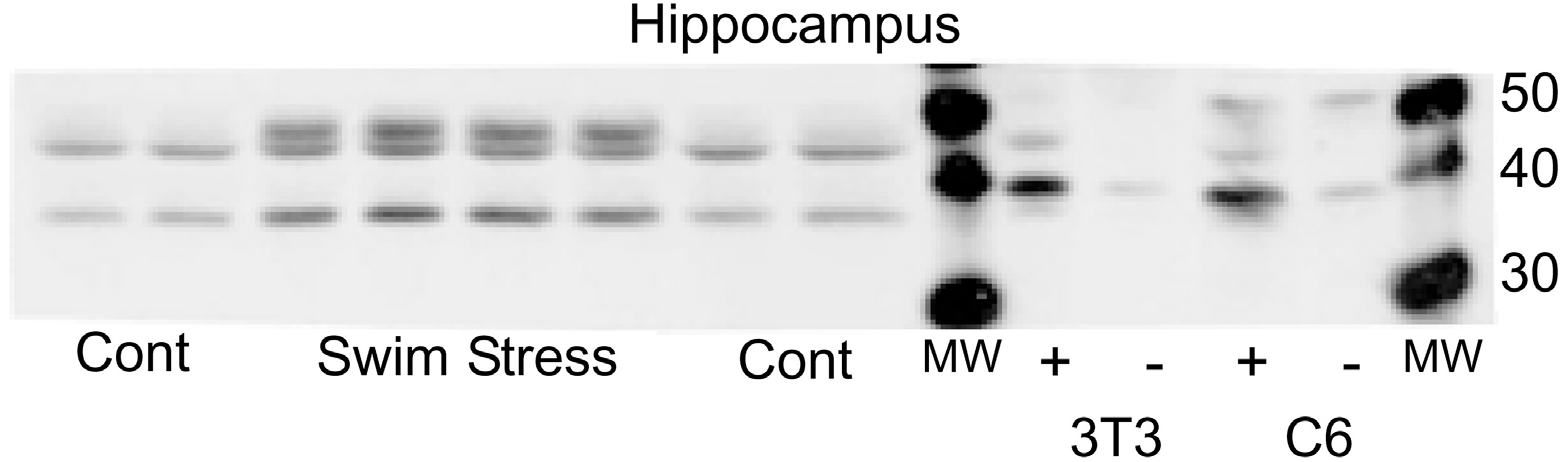
**Swim stress did not induce phosphorylation of MKK3/6. **Note the absence of a band for P-MKK3/6 at ~40 kD, either in control or swim stressed hippocampal lysates, whereas an induced band is visible in the positive control cell extracts. Also note the unknown ~37 and 48 kD proteins induced by swim stress. Other brain regions examined exhibited a similar pattern. 3T3, lysates of NIH/3T3 cells treated with (+) or without (-) UV radiation (40 mJ, 45 minutes recovery time); C6, lysates of C6 glioma cells treated with (+) or without (-) anisomycin (25 μg/ml, 30 min); MW, molecular weight markers.

In contrast to its effects on the Erk and JNK pathways, swim stress exhibited no effect on the phosphorylation of p38MAPK in any brain region, with the exception of a small but significant increase (55%) in the hippocampus (Figs. 5 and 6). As shown above, the major impact of swim stress was to activate the corresponding upstream MAPKKs of Erk and JNK, i.e., to increase P-MEK1/2 and P-MKK4, respectively. Since the major activators of p38MAPK are the closely related, dual specificity kinases phosphorylated MKK3 and MKK6 [39], we attempted to determine if swim stress increased the phosphorylation of this upstream kinase. Fig. 7 shows that the P-MKK3/6 antibody (CST # 9231) detected at least two swim stress-induced bands in the hippocampus, at ~37 and 48 kD. However, the expected molecular weight is ~40 kD; indeed, cell extracts of UV-treated NIH/3T3 and anisomycin-treated C6 glioma cells both exhibited induced bands at ~40 kD (Fig. 7). It seems likely, therefore, that basal levels of brain P-MKK3/6 are very low (*cf. *basal levels of P-MKK4, Fig. 3), and, unlike P-MEK1/2 and P-MKK4, are not induced by swim stress. Interestingly, use of a different P-MKK3/6 antibody (sc-7994-R, Santa Cruz) yielded very similar results, specifically, swim stress induced a large increase in an ~48 kD band (not shown). Other brain regions examined with the P-MKK3/6 antibody yielded the same pattern; no band at ~40 kD was evident (not shown). We considered the possibility that the swim-induced protein at ~48 kD might be P-MKK7, since that is the approximate molecular weight of this kinase. Moreover, MKK7, like MKK4, phosphorylates JNK [40]. Since the phosphopeptides used to raise the P-MKK4, P-MKK7 and P-MKK3/6 antibodies share a substantial degree of homology in their amino acid sequences, it was possible that the P-MKK3/6 antibody was detecting a swim-induced increase in P-MKK7. However, at a concentration of 1 ng/ml, neither the P-MKK4 nor the P-MKK7 phosphopeptides affected the protein band pattern observed using the P-MKK3/6 antibody, whereas the P-MKK3/6 phosphopeptide completely abolished all signals (data not shown). This appears to rule out the possibility that the 48 kD band is P-MKK7. The identities of the swim stress-induced bands observed with the P-MKK3/6 antibodies remain unknown.

In partial agreement with a previous report [27], swim stress induced significant increases in CREB phosphorylation in the hippocampus and amygdala (as well as in the striatum in the present study); however, we were unable to replicate reported P-CREB increases in neocortex and prefrontal cortex (Figs. 5 and 6). The reasons for this discrepancy are not apparent; however, while the same rat strain (Wistar) and swim protocol (15 min, followed by immediate sacrifice) were used, other differences (e.g., P-CREB antibody source, halothane vs. CO_2 _anesthesia, etc.) may account for the difference in results. Note, moreover, that the regional pattern of changes in P-CREB (Fig. 6) and P-Erk (Fig. 2) differed markedly. Whether this is due to a simple difference in time course or, more substantively, in activation mechanism, awaits further investigation.

### Time course of swim stress-induced MAPK kinase phosphorylation: onset and offset

**Figure 8:**
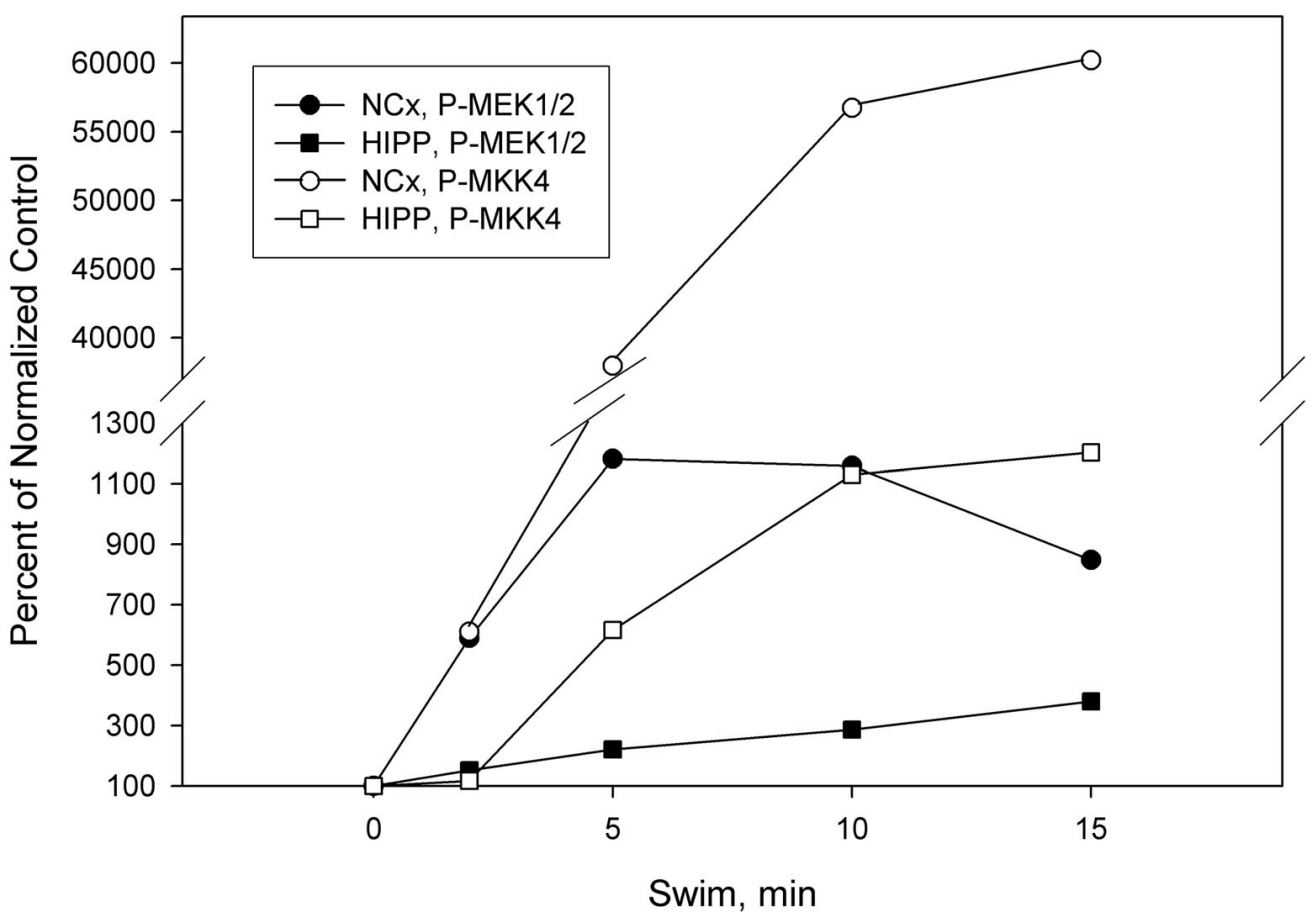
**Time-course of the onset of swim stress-induced increases in phosphorylation of MEK1/2 and MKK4. **Rats were sacrificed immediately upon termination of the indicated swim periods. Shown are the means of duplicate samples from a representative experiment. The experiment yielded similar results upon repetition.

**Figure 9:**
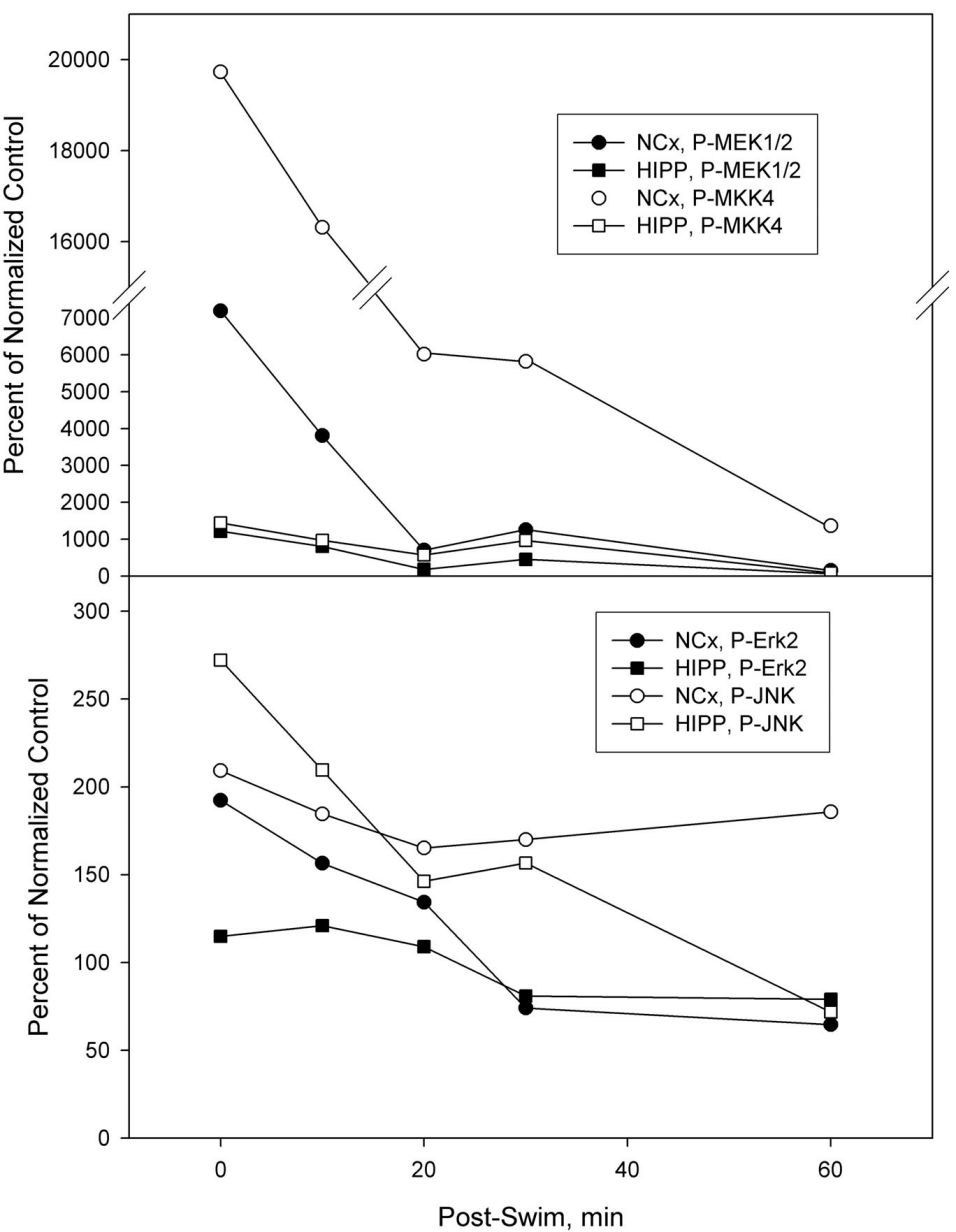
**Decline of swim-induced increases in phosphorylation of MAPK signaling proteins after stress termination. **Rats were subjected to a 15 min swim stress session and sacrificed at various times after swim termination. Top, time-course of the falloff in P-MEK1/2 and P-MKK4 phosphorylation in hippocampus (HIPP) and neocortex (NCx); bottom, time-course for P-Erk2 and P-JNK (all 3 isomers quantitated together). Shown are means of duplicate samples from a representative experiment that was repeated with similar results.

The effect of varying the duration of swim on the phosphorylation of MEK1/2 and MKK4 was examined in neocortex and hippocampus. The effect on both kinases gradually reached a maximum at 15 min (longer times were not assessed), except for the increase in P-MEK1/2 in neocortex, which peaked at 5 min (Fig. 8). The levels of P-MEK1/2 and P-MKK4 in both neocortex and hippocampus were maximal immediately after termination of swim stress (Fig. 9, top), declined gradually, and all except P-MKK4 in neocortex returned to basal levels by 60 min. The levels of the corresponding phosphorylated substrates of the MAPK kinases, P-Erk2 and P-JNK (combined P-JNK1 and P-JNK2/3) followed a very similar pattern of decline post-swim stress (Fig. 9, bottom), and all except neocortical P-JNK were at control levels by 60 min. Note that hippocampal P-Erk2 was unchanged at all times after swim stress, consistent with the results shown above in Figs. 1 and 2.

## Discussion

The results presented establish unambiguously that forced swim stress elicits a rapid and profound but relatively transient increase in the phosphorylation (and consequently the presumed activation) of the MEK-Erk and MKK4-JNK signaling pathways in, at the least, the five brain regions examined. These effects displayed specificity in that the MKK3/6-p38MAPK pathway was essentially unchanged. (Although in the experiment shown in Figs. 5 and 6, there was a very modest increase in P-p38MAPK that was restricted to the hippocampus, there was no significant change in this phosphoprotein in an independent replication of this experiment; data not shown). Our results may be compared to a very recent study on the effects of forced swim in mice, reported by Liu et al. [38] while the present study was nearing completion. Both studies are in agreement that swim stress elicits a very large increase in P-MKK4 levels throughout the brain, and that P-JNK levels are correspondingly elevated in the same brain regions. In addition, both studies agree that P-p38MAPK levels are unaffected. However, Liu et al. [38] did not determine the effects of swim stress on P-MEK1/2 levels (which we found were also markedly elevated: Figs. 1 and 2). Moreover, whereas Liu et al. [38] did not find any significant change in P-Erk levels, our studies indicated region-specific alterations in this kinase: increases in cortical areas (prefrontal and neocortical) and striatum, but not in the hippocampus or amygdala (Figs. 1 and 2). The reasons for the dissimilar results on P-Erk are not apparent, but it should be noted that the studies utilized different species (mice vs. rats), which may have contributed to the observed differences. Also, it is possible but unlikely that stress duration (30 min in the study of Liu et al. and 15 min here) accounts for the difference in results.

One of the most interesting aspects of the present results was the apparent discrepancy between the magnitude of the changes in the MAPKKs (P-MEK1/2 and P-MKK4) and the corresponding MAPKs (P-Erk and P-JNK). It is obvious from Figs. 2 and 4 that whereas swim stress elevated P-MEK1/2 and P-MKK4 levels dramatically, the effects on their substrates, P-Erk2 and P-JNK, respectively, were much more modest. (This difference appears to be much smaller in mice; [38]). One possible explanation is a temporal difference in the phosphorylation of the upstream MAPKKs and their downstream MAPK substrates. A gradual increase in P-Erk2 and P-JNK phosphorylation, subsequent to activation of MEK1/2 and MKK4, may have been obscured by the fact that animals were sacrificed immediately after termination of the swim. However, this is clearly not the case, as the time-course of the swim-induced increases in phosphorylation of the MAPKKs and MAPKs were very similar in both the hippocampus and neocortex (Fig. 9). Another explanation centers on potential differences in phosphorylation stoichiometry, i.e., levels of basal phosphorylation among the various phosphoproteins may vary greatly. Thus, one may posit that kinases (e.g., MEK1/2, MKK4) whose basal state of phosphorylation is very low may be subject to large increases, whereas those whose basal state is comparatively larger (e.g., Erk, JNK) demonstrate much smaller increases. For example, activation of tyrosine hydroxylase, the rate-limiting enzyme for catecholamine biosynthesis, is related to phosphorylation of the protein on at least three different serine sites [41]. The basal phosphorylation stoichiometry of these sites varies not only among the sites but also between brain regions [42]. Interestingly, haloperidol-induced phosphorylation of the sites, as visualized on immunoblots using phosphosite-specific antibodies, appears to be negatively related to the basal phosphorylation state [42]. To our knowledge, the basal phosphorylation stoichiometry of the various rat brain kinases examined in the present study are not known. However, a recent technique has been described [43] that may lend itself to estimating basal and swim stress-induced phosphorylation stoichiometry in various brain regions.

Nevertheless, differences in phosphorylation stoichiometry cannot explain why, in some brain regions (i.e., hippocampus and amygdala, Figs. 1 and 2), there were no discernable increases in P-Erk after swim stress, despite large increases in P-MEK1/2. Activated MAPKKs, unlike their upstream activators or downstream targets, are known to exhibit narrow substrate specificity [13]. Indeed, Erk1/2 activity is believed to be exclusively regulated by MEK1/2 [15]. Thus, a large increase in MEK1/2 phosphorylation/activation would be expected to translate into substantial phosphorylation/activation of Erk. However, the activity of P-MEK1/2 is regulated by a negative feedback mechanism involving activation of protein phosphatase(s) that rapidly dephosphorylate P-Erk1/2 [44]. A large number of potential phosphatases have been implicated in the regulation of Erk phosphorylation [44]; consequently, identifying a specific phosphatase that may be responsible for such an effect was beyond the scope of the present study. Recently, a detailed kinetic analysis of eleven different protein phosphatases implicated three specific phosphatases as the most likely mediators of Erk2 dephosphorylation, including mitogen-activated protein kinase phosphatase 3 (MKP-3) and protein phosphatase 2A (PP2A) [45]. It might be instructive therefore in a future study to assess the activity of these protein phosphatases in tissue lysates. A final alternative hypothesis is that swim stress activates a mechanism that serves to uncouple MEK1/2 phosphorylation (fully or in part) from activation of Erk. That such uncoupling is possible has been reported in mammalian cells undergoing mitosis [46], but the mechanism deduced in that instance is highly unlikely to apply in the present case; nevertheless, the possibility is worthy of consideration.

In a previous communication we reported that 30 min of restraint stress elicited modest (1.6–2.5-fold) increases in P-Erk levels in hippocampus and prefrontal and cingulate cortex [37]. As for swim stress, P-p38MAPK was not altered. In contrast to the effects of swim stress, P-JNK levels were not altered by restraint. That study did not assess changes in any of the MAPKKs, limiting further comparisons. However, in a preliminary experiment we found that restraint stress increased P-MKK4 levels in both the hippocampus and striatum, but the effects were more modest than after swim stress (data not shown). Interestingly, Liu et al. [38] reported that restraint, like swim, elevated both P-MKK4 and P-JNK levels in mice, but the effects in the former stress model were smaller than in the latter. However, P-Erk and P-p38MAPK levels were unchanged in both restrained and swim stressed mice. It thus appears that both the rodent species and type of stress may be important determinants of the specific regional changes occurring in the various kinases. Additional studies will be required to sort out the influence of a variety of factors on the phosphorylation pattern of the MAPK signaling pathways in the brain regions of different animal species. In particular, restraint and swim differences in ambient temperature and physical activity may impact the results. Finally, because these two forms of stress yield different patterns of effects on MAPKs in brain, we can ask which MAPKs (and which brain regions) are the more relevant to stress effects, i.e., which stress model is more appropriate. Answering this question will require correlating the behavioral and MAPK sequelae of different stress treatments (see below).

The nature of the extracellular signaling molecule(s) that are recruited upon subjecting animals to stress are unknown. The pathways leading from stimulation of a particular receptor on the cell surface to activation of MAPK signaling cascades are diverse and complex [13,14,47-50]. For example, Erk1 and Erk2 can be activated by a variety of extracellular signaling molecules, including growth factors, hormones and neurotransmitters [48-52]. Calcium influx in particular has gained currency as potentially of prime importance [53]. As mentioned earlier, stress is well known to activate *c-fos *gene expression) [21-24], and such activation in neurons can occur, at least in part, because of an increase in Erk activity *via *NMDA glutamate receptor-stimulated calcium influx [54-56]. Stress also activates CREB phosphorylation [27], which may be mediated at least in part by NMDA receptor activation [57]. Thus, it is attractive to speculate that NMDA receptor activation (at least in some brain regions) may be the initial trigger leading to activation of the Erk pathway (calcium influx may also lead to activation of the JNK pathway via other signaling mechanisms; see [53]). NMDA receptors themselves are subject to regulation by phosphorylation on their NR1, NR2A and NR2B subunits [58-60]. Interestingly, dopamine D1 receptor-mediated CREB phosphorylation appears to involve NMDA NR1 subunit phosphorylation [61]. In preliminary experiments we have found that swim stress increased P-NR1 levels on immunoblots of the hippocampus, neocortex and striatum (data not shown), suggesting that stress-induced activation of the MEK-Erk pathway may involve NMDA receptor-mediated calcium influx. Further studies will be directed toward explicating the differential effects of swim stress on the degree of phosphorylation of the MAPKKs vs. the MAPKs, assessing NMDA receptor involvement, and determining whether (and which) MAPKK pathways participate in some behavioral sequelae of repeated swim stress treatment (e.g., immobility and analgesia).

## Conclusions

Acute swim stress initiated a rapid increase in phosphorylation of the MAPKKs MEK1/2 and MKK4 in hippocampus, neocortex, prefrontal cortex, amygdala and striatum. Concomitantly, their corresponding substrates Erk and JNK were also phosphorylated, but not always in register with the changes in the MAPKKs. Moreover, the magnitude of the increase in phosphorylation of MEK1/2 and MKK4 was much greater than for their cognate substrates. While it is clear that stressors such as forced swim and restraint activate these signaling pathways, much work will be required to define the mechanism(s) responsible for these effects, and to relate potential alterations in the activity of these molecules to stress-related influences on the development of anxiety and depressive disorders.

## Methods

### Animals

Male Wistar rats (200–300 g; Taconic Farms, Germantown, NY) were used throughout. Rats were housed 2/cage and maintained on a 12 h light/dark cycle with food and water ad libitum; they were acclimated to handling and transportation for 2–3 weeks before being used in experiments. All experimental procedures were carried out in accordance with the NIH *Guide for the Care and Use of Laboratory Animals*, and were approved by the NYU School of Medicine Institutional Animal Care and Use Committee.

### Materials

Precast 10% polyacrylamide gels were obtained from Cambrex BioScience, Rockland, ME. Complete^® ^protease inhibitor cocktail was from Roche Molecular Biochemicals, Indianapolis, IN and colored molecular weight markers were purchased from Bio-Rad, Hercules, CA. Biotinylated protein ladder marker and anti-biotin were from Cell Signaling Technology (CST), Beverly, MA. West Pico Super Signal enhanced chemiluminescence (ECL) reagent was obtained from Pierce Biotechnology, Rockford, IL, and Re-Blot Plus stripping buffer was from Chemicon International, Temecula, CA. All primary antibodies were rabbit polyclonal except as noted. Because suppliers often offer more than a single antibody for the same antigen, catalog numbers are specified in parentheses. P-Erk (monoclonal, sc-7383), pan Erk (sc-93) and pan MKK4/SEK1 (sc-964) were all from Santa Cruz Biotechnologies, Santa Cruz, CA. The following antibodies were obtained from CST: P-MEK1/2 (9121), pan MEK1/2 (9122), P-MKK4/SEK1 (9151), P-JNK (monoclonal, 9255), pan JNK (9252), P-MKK3/6 (9231), pan CREB (9192) and pan p38MAPK (9212). P-CREB (06-519) was from Upstate Biotechnology, Inc. (Waltham, MA), while P-p38 was either from CST (9212) or Chemicon (AB3828). Control cell extracts for P-MKK3/6 were obtained from CST: +/- UV-treated NIH/3T3 cell lysates (9233) and +/- anisomycin-treated C6 glioma cell lysates (9213).

### Stress procedure

Groups of rats (n = 4–6) were subjected to forced swim stress in a round glass tank (24 cm W × 44 cm H) filled to a depth of 30 cm with water (25 ± 1°C). In most experiments rats were forced to swim for 15 min and sacrificed by decapitation immediately after narcotization with carbon dioxide for 20 sec. In some experiments the duration of swim stress (2–15 min) or the post-swim interval (0–60 min) was varied as described in the legends to Figures. Control rats were not treated and were sacrificed in parallel as above.

### Tissue dissection

Brains were rapidly removed, briefly chilled in saline and placed in a stainless steel brain matrix (Activational Systems, Warren, MI). Sections of 1–2 mm in thickness were cut, frozen quickly on dry ice, and brain areas of interest (prefrontal cortex, neocortex, amygdala and striatum) were micropunched using the atlas of Paxinos and Watson [62] as a guide. Hippocampus was dissected freehand from the remaining portion of the brain (approximately posterior to bregma -3.80). All samples were stored at -80°C until processed.

### Tissue processing

Tissues were mechanically disrupted, in glass homogenizers using a Teflon pestle, in 10–20 volumes of buffer (50 mM Tris-HCl, pH 7.4 containing 300 mM NaCl, 1% Nonidet P-40, 10% glycerol, 1 mM EDTA, 1 mM Na_3_VO_4_, 1 mM NaF, 0.5 μM okadaic acid and Complete^® ^protease inhibitor cocktail). After centrifugation at 30,000 *g *for 20 min, lysates were mixed with 5X sodium dodecyl sulfate (SDS) sample buffer, boiled for 5 min and stored at -80°C. Protein content of lysates was determined using the bicinchoninic acid assay kit (Pierce).

### Protein separation and immunoblotting

Proteins (10–40 μg/lane) were separated by SDS-PAGE on precast polyacrylamide gels. Gels were also loaded with colored molecular weight markers to assess electrophoretic transfer, and biotinylated protein ladder marker to estimate molecular weights of bands of interest. Following electrophoretic transfer to nitrocellulose, blots were incubated in blocking buffer (5% nonfat dry milk in Tris-buffered saline containing 0.05% Tween-20 (TBST)) for 1 hr at room temperature (RT), washed 3 × 10 min in TBST and incubated with primary phospho-specific antibodies overnight at 4°C (monoclonal antibodies in 5% milk/TBST, polyclonal antibodies in 5% BSA/TBST). Blots were washed 3× in TBST, incubated with the appropriate secondary antibody plus anti-biotin for 1 hr at RT, washed again, treated with ECL reagent and exposed to film. Blots were then stripped by incubation for 15 min at RT with Re-Blot Plus, re-blocked, washed and incubated for 1 hr at RT with the corresponding pan antibody which recognizes total antigen protein (phosphorylated and nonphosphorylated). This was followed by incubation for 1 hr at RT with the appropriate secondary antibody plus anti-biotin. Antigens were again visualized by treatment with ECL reagent and exposure to film.

### Immunoblot analysis

A computerized image analysis system (MCID-M4, Imaging Research, St. Catherines, Ontario, Canada) was utilized to analyze immunoblots. Image analysis was carried out as described previously [63]. For each blot, relative phosphoprotein levels were calculated from the ratio of absorbance of the phosphoprotein/pan protein to correct for small differences in protein loading. Differences between experimental and control conditions were obtained by comparison to the normalized control ratio (arbitrarily set at 100).

### Statistical analysis

Control and swim-stressed groups were compared using Student's *t *test (SigmaStat, ver. 2.03, SPPS, Inc., Chicago, IL).

## Authors' contributions

C-PS conducted the majority of the immunoblotting studies. YT performed some of the experiments and quantitated many of the films using image analysis. CS assisted in the design of some of the experiments, carried out the MKK3/6 experiments, and assisted in the writing of the manuscript. EM designed the study, supervised and facilitated the experiments, analyzed the data and wrote the manuscript.

## References

[B1] Post RM (1992). Transduction of psychosocial stress into the neurobiology of recurrent affective disorder. Am J Psychiatry.

[B2] Kendler KS, Thornton LM, Gardner CO (2001). Genetic risk, number of previous depressive episodes, and stressful life events in predicting onset of major depression. Am J Psychiatry.

[B3] Garcia R (2002). Stress, synaptic plasticity, and psychopathology. Rev Neurosci.

[B4] Owens MJ (2004). Selectivity of antidepressants: from the monoamine hypothesis of depression to the SSRI revolution and beyond. J Clin Psychiatry.

[B5] Shirayama Y, Chen ACH, Nakagawa S, Russell DS, Duman RS (2002). Brain-derived neurotrophic factor produces antidepressant effects in behavioral models of depression. J Neurosci.

[B6] Cammarota M, Bevilaqua LR, Ardenghi P, Paratcha G, Levi de Stein M, Izquierdo I, Medina JH (2000). Learning-associated activation of nuclear MAPK, CREB and Elk-1, along with Fos production, in the rat hippocampus after a one-trial avoidance learning: abolition by NMDA receptor blockade. Brain Res Mol Brain Res.

[B7] Davis S, Vanhoutte P, Pages C, Caboche J, Laroche S (2000). The MAPK/ERK cascade targets both Elk-1 and cAMP response element-binding protein to control long-term potentiation-dependent gene expression in the dentate gyrus in vivo. J Neurosci.

[B8] Schafe GE, Atkins CM, Swank MW, Bauer EP, Sweatt JD, LeDoux JE (2000). Activation of ERK/MAP kinase in the amygdala is required for memory consolidation of Pavlovian fear conditioning. J Neurosci.

[B9] Manji HK, Drevets WC, Charney DS (2001). The cellular neurobiology of depression. Nat Med.

[B10] Flood DG, Finn JP, Walton KM, Dionne CA, Contreras PC, Miller MS, Bhat RV (1998). Immunolocalization of the mitogen-activated protein kinases p42MAPK and JNK1, and their regulatory kinases MEK1 and MEK4, in adult rat central nervous system. J Comp Neurol.

[B11] Ortiz J, Harris HW, Guitart X, Terwilliger RZ, Haycock JW, Nestler EJ (1995). Extracellular signal-regulated protein kinases (ERKs) and ERK kinase (MEK) in brain: regional distribution and regulation by chronic morphine. J Neurosci.

[B12] Schaeffer HJ, Weber MJ (1999). Mitogen-activated protein kinases: Specific messages from ubiquitous messengers. Mol Cell Biol.

[B13] Houslay MD, Kolch W (2000). Cell-type specific integration of cross-talk between extracellular signal-regulated kinase and cAMP signaling. Mol Pharmacol.

[B14] Grewal SS, York RD, Stork PJ (1999). Extracellular-signal-regulated kinase signalling in neurons. Curr Opin Neurobiol.

[B15] Sweatt JD (2001). The neuronal MAP kinase cascade: a biochemical signal integration system subserving synaptic plasticity and memory. J Neurochem.

[B16] Murray B, Alessandrini A, Cole AJ, Yee AG, Furshpan EJ (1998). Inhibition of the p44/42 MAP kinase pathway protects hippocampal neurons in a cell-culture model of seizure activity. Proc Natl Acad Sci USA.

[B17] Alessandrini A, Namura S, Moskowitz MA, Bonventre JV (1999). MEK1 protein kinase inhibition protects against damage resulting from focal cerebral ischemia. Proc Natl Acad Sci USA.

[B18] Gioia M, Galbiati S, Rigamonti L, Moscheni C, Gagliano N (2001). Extracellular signal-regulated kinases 1 and 2 phosphorylated neurons in the tele- and diencephalon of rat after visceral pain stimulation: an immunocytochemical study. Neurosci Lett.

[B19] Bhat RV, Engber TM, Finn JP, Koury EJ, Contreras PC, Miller MS, Dionne CA, Walton KM (1998). Region-specific targets of p42/p44MAPK signaling in rat brain. J Neurochem.

[B20] Oh SW, Ahn YM, Kang UG, Kim YS, Park JB (1999). Differential activation of c-Jun N-terminal protein kinase and p38 in rat hippocampus and cerebellum after electroconvulsive shock. Neurosci Lett.

[B21] Figueiredo HF, Dolgas CM, Herman JP (2002). Stress activation of cortex and hippocampus is modulated by sex and stage of estrus. Endocrinology.

[B22] Stamp JA, Herbert J (1999). Multiple immediate-early gene expression during physiological and endocrine adaptation to repeated stress. Neuroscience.

[B23] Deutch AY, Lee MC, Gillham MH, Cameron DA, Goldstein M, Iadarola MJ (1991). Stress selectively increases fos protein in dopamine neurons innervating the prefrontal cortex. Cereb Cortex.

[B24] Melia KR, Ryabinin AE, Schroeder R, Bloom FE, Wilson MC (1994). Induction and habituation of immediate early gene expression in rat brain by acute and repeated restraint stress. J Neurosci.

[B25] Finkbeiner S, Tavazoie SF, Maloratsky A, Jacobs KM, Harris KM, Greenberg ME (1997). CREB: a major mediator of neuronal neurotrophin responses. Neuron.

[B26] Treisman R (1995). Journey to the surface of the cell: Fos regulation and the SRE. EMBO J.

[B27] Bilang-Bleuel A, Rech J, De Carli S, Holsboer F, Reul JM (2002). Forced swimming evokes a biphasic response in CREB phosphorylation in extrahypothalamic limbic and neocortical brain structures in the rat. Eur J Neurosci.

[B28] Watanabe Y, Sakai RR, McEwen BS, Mendelson S (1993). Stress and antidepressant effects on hippocampal and cortical 5-HT_1A _and 5-HT_2 _receptors and transport sites for serotonin. Brain Res.

[B29] Haddjeri N, Blier P, De Montigny C (1998). Long-term antidepressant treatments result in a tonic activation of forebrain 5-HT_1A _receptors. J Neurosci.

[B30] Thierry AM, Tassin JP, Blanc G, Glowinski J (1976). Selective activation of mesocortical DA system by stress. Nature.

[B31] Clement H-W, Schäfer F, Ruwe C, Gemsa D, Wesemann W (1993). Stress-induced changes of extracellular 5-hydroxyindoleacetic acid concentrations followed in the nucleus raphe dorsalis and the frontal cortex of the rat. Brain Res.

[B32] Gruen RJ, Wenberg K, Elahi R, Friedhoff AJ (1995). Alterations in GABA_A _receptor binding in the prefrontal cortex following exposure to chronic stress. Brain Res.

[B33] Lavielle S, Tassin JP, Thierry AM, Blanc G, Herve D, Barthelemy C, Glowinski J (1979). Blockade by benzodiazepines of the selective high increase in dopamine turnover induced by stress in mesocortical dopaminergic neurons of the rat. Brain Res.

[B34] Davis M, Hitchcock JM, Bowers MB, Berridge CW, Melia KR, Roth RH (1994). Stress-induced activation of prefrontal cortex dopamine turnover: Blockade by lesions of the amygdala. Brain Res.

[B35] Lombardo KA, Herringa RJ, Balachandran JS, Hsu DT, Bakshi VP, Roseboom PH, Kalin NH (2001). Effects of acute and repeated restraint stress on corticotropin-releasing hormone binding protein mRNA in rat amygdala and dorsal hippocampus. Neurosci Lett.

[B36] Kawahara H, Yoshida M, Yokoo H, Nishi M, Tanaka M (1993). Psychological stress increases serotonin release in the rat amygdala and prefrontal cortex assessed by in vivo microdialysis. Neurosci Lett.

[B37] Meller E, Shen C, Nikolao TA, Jensen C, Tsimberg Y, Chen J, Gruen RJ (2003). Region-specific effects of acute and repeated restraint stress on the phosphorylation of mitogen-activated protein kinases. Brain Res.

[B38] Liu YF, Bertram K, Perides G, McEwen BS, Wang D (2004). Stress induces activation of stress-activated kinases in the mouse brain. J Neurochem.

[B39] Raingeaud J, Whitmarsh AJ, Barrett T, Derijard B, Davis RJ (1996). MKK3- and MKK6-regulated gene expression is mediated by the p38 mitogen-activated protein kinase signal transduction pathway. Mol Cell Biol.

[B40] Tournier C, Whitmarsh AJ, Cavanagh J, Barrett T, Davis RJ (1999). The MKK7 gene encodes a group of c-Jun NH2-terminal kinase kinases. Mol Cell Biol.

[B41] Haycock JW (1990). Phosphorylation of tyrosine hydroxylase in situ at serine 8, 19, 31, and 40. J Biol Chem.

[B42] Salvatore MF, Garcia-Espana A, Goldstein M, Deutch AY, Haycock JW (2000). Stoichiometry of tyrosine hydroxylase phosphorylation in the nigrostriatal and mesolimbic systems in vivo: effects of acute haloperidol and related compounds. J Neurochem.

[B43] Mîinea CP, Lienhard GE (2003). Stoichiometry of site-specific protein phosphorylation estimated with phosphopeptide-specific antibodies. BioTechniques.

[B44] Haneda M, Sugimoto T, Kikkawa R (1999). Mitogen-activated protein kinase phosphatase: a negative regulator of the mitogen-activated protein kinase cascade. Eur J Pharmacol.

[B45] Zhou B, Wang ZX, Zhao Y, Brautigan DL, Zhang ZY (2002). The specificity of extracellular signal-regulated kinase 2 dephosphorylation by protein phosphatases. J Biol Chem.

[B46] Harding A, Giles N, Burgess A, Hancock JF, Gabrielli BG (2003). Mechanism of mitosis-specific activation of MEK1. J Biol Chem.

[B47] Vanhoutte P, Barnier JV, Guibert B, Pagès C, Besson MJ, Hipskind RA, Caboche J (1999). Glutamate induces phosphorylation of Elk-1 and CREB, along with c-*fos *activation, via an extracellular signal-regulated kinase-dependent pathway in brain slices. Mol Cell Biol.

[B48] Graves JD, Krebs EG (1999). Protein phosphorylation and signal transduction. Pharmacol Ther.

[B49] Cobb MH (1999). MAP kinase pathways. Prog Biophys Mol Biol.

[B50] Gutkind JS (1998). The pathways connecting G protein-coupled receptors to the nucleus through divergent mitogen-activated protein kinase cascades. J Biol Chem.

[B51] Fukunaga K, Miyamoto E (1998). Role of MAP kinase in neurons. Mol Neurobiol.

[B52] Schlessinger J (2000). Cell signaling by receptor tyrosine kinases. Cell.

[B53] Curtis J, Finkbeiner S (1999). Sending signals from the synapse to the nucleus: possible roles for CaMK, Ras/ERK, and SAPK pathways in the regulation of synaptic plasticity and neuronal growth. J Neurosci Res.

[B54] Xia Z, Dudek H, Miranti CK, Greenberg ME (1996). Calcium influx via the NMDA receptor induces immediate early gene transcription by a MAP kinase/ERK-dependent mechanism. J Neurosci.

[B55] English JD, Sweatt JD (1997). A requirement for the mitogen-activated protein kinase cascade in hippocampal long term potentiation. J Biol Chem.

[B56] Mao L, Tang Q, Samdani S, Liu Z, Wang JQ (2004). Regulation of MAPK/ERK phosphorylation via ionotropic glutamate receptors in cultured rat striatal neurons. Eur J Neurosci.

[B57] Nijholt I, Blank T, Ahi J, Spiess J (2002). In vivo CREB phosphorylation mediated by dopamine and NMDA receptor activation in mouse hippocampus and caudate nucleus. Gene Expr Patterns.

[B58] Swope SL, Moss SJ, Raymond LA, Huganir RL (1999). Regulation of ligand-gated ion channels by protein phosphorylation. Adv Second Messenger Phosphoprotein Res.

[B59] Hisatsune C, Umemori H, Inoue T, Michikawa T, Kohda K, Mikoshiba K, Yamamoto T (1997). Phosphorylation-dependent regulation of N-methyl-D-aspartate receptors by calmodulin. J Biol Chem.

[B60] Tingley WG, Ehlers MD, Kameyama K, Doherty C, Ptak JB, Riley CT, Huganir RL (1997). Characterization of protein kinase A and protein kinase C phosphorylation of the N-methyl-D-aspartate receptor NR1 subunit using phosphorylation site-specific antibodies. J Biol Chem.

[B61] Dudman JT, Eaton ME, Rajadhyaksha A, Macias W, Taher M, Barczak A, Kameyama K, Huganir R, Konradi C (2003). Dopamine D1 receptors mediate CREB phosphorylation via phosphorylation of the NMDA receptor at Ser897-NR1. J Neurochem.

[B62] Paxinos G, Watson C (1998). The Rat Brain in Stereotaxic Coordinates.

[B63] Meller E, Li H, Carr KD, Hiller JM (2000). 5-Hydroxytryptamine_1A _receptor-stimulated [^35^S]GTPγS binding in rat brain: Absence of regional differences in coupling efficiency. J Pharmacol Exp Ther.

